# Predicting Histological Healing and Recurrence in Ulcerative Colitis by Assessing Mucosal Vascular Pattern Under Narrow-Band Imaging Endoscopy

**DOI:** 10.3389/fmed.2022.869981

**Published:** 2022-06-30

**Authors:** Tao He, Lei Zong, Peng Pan, Shanming Sun, Hongmei Qu

**Affiliations:** ^1^Department of Clinical Medical College, Weifang Medical University, Weifang, China; ^2^Department of Gastroenterology, The First Affiliated Hospital of Weifang Medical College, Weifang People's Hospital, Weifang, China

**Keywords:** high-definition endoscopy, ulcerative colitis, recurrence, narrow-band optical imaging, histological healing

## Abstract

This study investigated the predictive value of narrow-band imaging (NBI) endoscopic staging of different mucosal vascular patterns (MVPs) in patients with ulcerative colitis (UC) for histological healing or clinical recurrence of patients with UC. A total of 124 patients with UC in clinical remission attending the First Affiliated Hospital of Weifang Medical College were included in the study and underwent NBI colonoscopy. Inflammatory activity was assessed in the intestine using the Mayo endoscopic score (MES) and the MVP. Mucosal inflammation was histologically graded using the Nancy index (NI). The colons of 124 patients with UC were staged according to NBI endoscopic MVP staging criteria. The differences between NBI colonoscopy MVP typing and white light endoscopic MES in assessing histological healing (HH) were statistically significant (*p* < 0.001), and there was a moderate correlation between MES and the degree of HH (*r* = 0.471, *p* < 0.001). In addition, there was a significant correlation between the severity of mucosal activity determined by white light endoscopy (WLE) and MVP staging (*r* = 0.811, *p* < 0.001). The differences between NBI endoscopic MVP staging and white light endoscopic MES in assessing UC recurrence were statistically significant (*p* < 0.001). Spearman's correlation analysis showed a moderate correlation between NBI endoscopic MVP staging and clinical recurrence. NBI endoscopic MVP staging can predict HH and clinical recurrence status better than WLE.

## Introduction

Inflammatory bowel disease, such as Crohn's disease and ulcerative colitis (UC), is a chronic inflammatory disorder of the gastrointestinal tract. Multiple variables are thought to be implicated in the pathogenesis *via* the activation of mucosal immune responses in gut-associated lymphoid tissues, such as intestinal microbiota, dietary antigens, and other environmental elements (1). Long-term treatment goals have changed from symptom control to endoscopic mucosal healing with clinical remission in recent years (2). However, several reports have shown that mucosa that had seemingly healed still had a slight histologically defined inflammation and the possibility of relapse (3). Therefore, histological remission becomes the next therapy objective after endoscopic mucosal healing and has given rise to the new concept of “disease clearance,” which includes clinical, endoscopic, and microscopic remission (4).

Endoscopy is essential for evaluating disease activity and the efficacy of treatment interventions in UC. There are several different scoring systems for the endoscopic evaluation of UC severity. The Mayo scoring system is commonly used, and Mayo scores of both 0 and 1 are defined as mucosal healing (5). Narrow-band imaging (NBI) is an image-enhanced endoscope-based approach to enhance the fine structure of the mucosa without the use of dyes. In addition, NBI can visualize subtle changes in the mucosa and the distribution of abnormal blood vessels (6). Accordingly, NBI colonoscopy might be a valuable tool to assess mucosal angiogenesis according to the mucosal vascular pattern (MVP) in UC (7). However, there seem to be few investigations into the relationship between MVP based on NBI colonoscopy and histological assessment in UC.

This study aims to establish the performance of and relationship between NBI and white light endoscopy (WLE) assessments in patients with UC and their association with histological healing (HH). Furthermore, we wanted to evaluate whether endoscopic pictures of MVP obtained by NBI are a predictor of clinical outcomes.

## Materials and Methods

### Patients

This study was conducted at the First Affiliated Hospital of Weifang Medical College between January 2018 and December 2020 in patients with an established diagnosis of UC. The extent of UC was determined by colonoscopy, and the UC disease activity was assessed according to the Mayo score. Patients were examined by NBI colonoscopy with biopsy and had signed informed consent for colonoscopy.

### Instrument

Each patient underwent a colonoscopy with an endoscope (CF-H290AI; Olympus, Tokyo, Japan), using a prototype of the NBI system (Elvis CV-290; Olympus, Tokyo, Japan).

### Study Procedure

Total colonoscopy in 124 patients with UC was performed with WLE and NBI to observe the mucosa of each segment of the colorectum. The colorectum was divided into six segments for assessment, defined as the cecum, ascending colon, transverse colon, descending colon, sigmoid colon, and rectum. From the same lesions observed by WLE and NBI, targeted biopsies of the most inflamed area assessed by colonoscopy were obtained and analyzed.

We first defined the NBI findings into three categories, as described below. The WLE and NBI findings were compared, with the white light images being divided into two groups: mucosal healing or not. We first assessed this to determine if the standard white light classification can predict NBI findings, which seemed only to be able to indicate mucosal healing. Then, the pathological results from all of the biopsy specimens were compared with the NBI findings to assess the relationship between imaging and pathological activity.

The relationships between the NBI findings at initial ileocolonoscopy and 1-year follow-up clinical outcomes were assessed to reveal the different prognoses for the NBI findings.

Patients were followed until the end of the study in 2020 or recurrence. Recurrence was defined as a Mayo endoscopic score (MES) ≥ 2, therapy to induce remission, hospitalization, or colectomy (8).

### Endoscopic Assessment

All WLE and NBI images from all target areas were randomly assigned to two experienced endoscopists who were unfamiliar with the clinical data. If there was a disagreement, an agreement was reached through negotiation. The endoscopic activity under WLE was classified into four degrees of severity, from 0 to 3 (0: inactive, 1: mild, 2: moderate, and 3: severe) according to MES (9).

**Figure 1:**
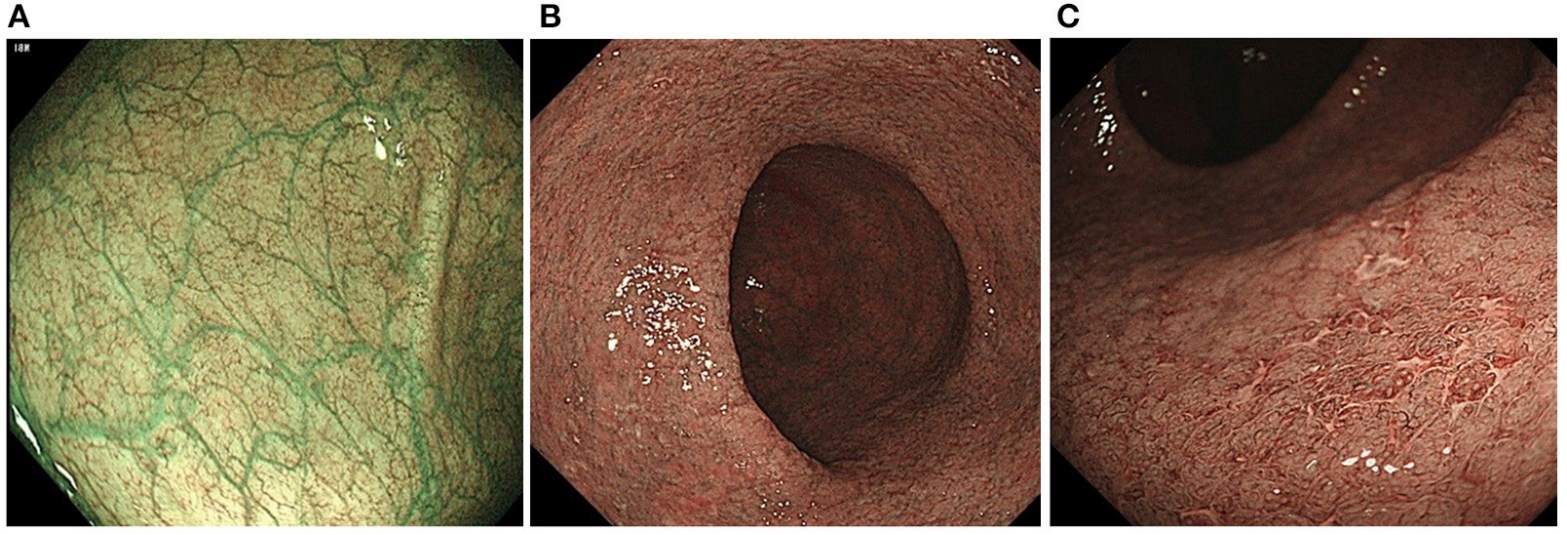
Classification of the narrow-band imaging (NBI) findings: **(A)** clear; **(B)** obscure; and **(C)** absent.

When NBI colonoscopy demonstrated clear intramucosal capillaries, the MVP was defined as clear. When NBI failed to reveal a clear vascular network or blurry image, it was regarded as obscure. In areas where the intramucosal vessels were invisible under NBI, the MVP was regarded as absent. Based on this protocol, the MVPs were classified into three types: clear, obscure, or absent (10) (Figure 1).

### Histological Assessment

**Figure 2:**
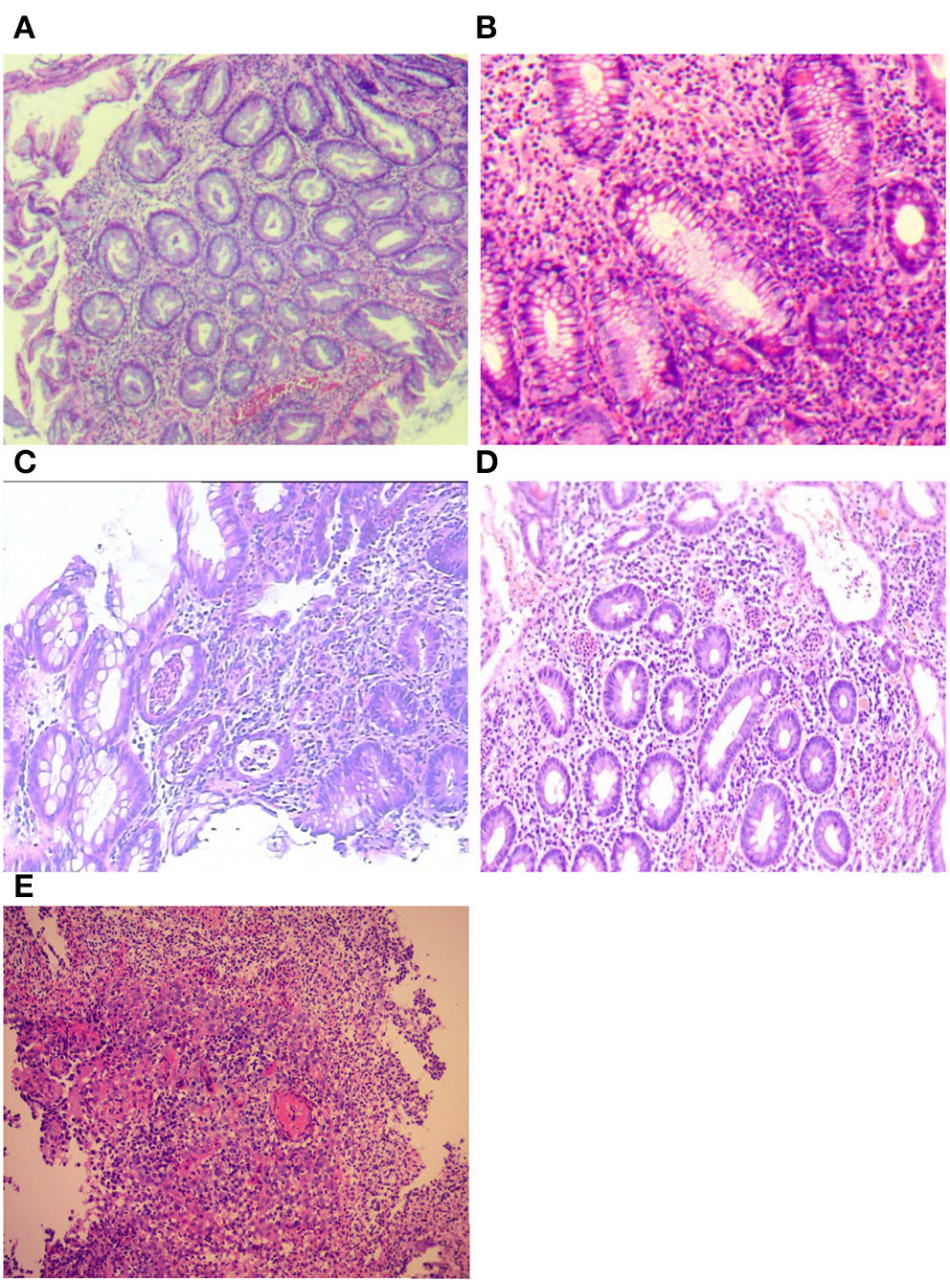
Histological assessment of Nancy index (NI): **(A)** no significant histological disease (Grade 0); **(B)** chronic inflammatory infiltrate with no acute inflammatory infiltrate (Grade 1); **(C)** mildly active disease (Grade 2); **(D)** moderately active disease (Grade 3); and **(E)** severely active disease (Grade 4).

All histologic specimens from colonoscopy were reviewed by a single senior gastrointestinal pathologist who was blinded to the outcome of interest. The site with the most histologically active disease was scored according to the Nancy index (NI) (11). The individual components of the NI were recorded for each patient: no significant histological disease (Grade 0), chronic inflammatory infiltrate with no acute inflammatory infiltrate (Grade 1), mildly active disease (Grade 2), moderately active disease (Grade 3), or severely active disease (Grade 4). We defined histological inactivity for grades 0 and 1 and histological activity for grades 2–4 (11) (Figure 2). The worst score obtained was considered if different NI classifications were obtained from the different segments of the colon.

### Outcomes

Clinical outcomes at follow-up were specified as (1) hospitalization as a result of UC relapse, (2) colectomy, or (3) initiation or changes in medical therapy, such as steroids, immunomodulators, and biologics, due to UC relapse. The recorded notes of all patients were reviewed to record these clinical outcome events, with telephone calls made to clarify any uncertainty, 12 months after the colonoscopy procedure.

### Statistical Analysis

All statistical analyses were performed using the IBM SPSS Statistics 26 software package (IBM, New York, NY, USA). Continuous data are presented using the mean and SD, whereas proportions and percentages are used to describe discrete variables. Wilcoxon Mann–Whitney *U*-tests and Kruskal–Wallis tests were used for non-parametric values, and one-way analysis of variance (ANOVA) was used for the parametric values. For correlations, Spearman's rank correlation coefficient was used. The predictive power of the diagnostic model was evaluated using receiver operating characteristics (ROCs) analysis, and the cut-off value was obtained when the Youden index reached the maximum. Sensitivity, specificity, and area under the ROC curve (AUC) were used to assess the prediction accuracy. A two-tailed *p*-value < 0.05 was defined as statistically significant. A biomedical statistician reviewed the statistical methods of this study.

## Results

### Clinical Characteristics of Patients

We obtained clinical data, endoscopic scores, and histological scores from 124 patients with UC (52 men [M]; 41.9%) who underwent colonoscopy and were recruited for this study. Clinical characteristics are shown in Table 1. Based on the colonoscopy and Mayo score, the extent and activity of the disease were determined.

**Table 1:** Clinical characteristics of patients with ulcerative colitis (UC).

**Clinical characteristics**	***N* (%)**
Patients with UC	124
**Sex**	
Male	52 (41.9%)
Female	72 (58.1%)
Age (years), median (range)	48 (25–83)
Disease duration (years), median (range)	5 (1–30)
**Maximum extent of UC**	
Extensive colitis	26 (21.0%)
Left-sided colitis	54 (43.5%)
Proctitis	44 (35.5%)
**Mayo endoscopic subscore**	
MES ≤ 1	35 (28.2%)
MES = 2	60 (48.4%)
MES = 3	37 (23.4%)

*UC, ulcerative colitis*.

### NBI Colonoscopy Findings in UC

A total of 124 colorectal segments from 124 patients with UC were analyzed. Under NBI colonoscopy, 37 segments were determined as having a clear MVP, 57 segments were judged as having an obscure MVP, and 30 segments had an absent MVP (Table 2).

**Table 2:** Colonoscopy findings in patients with UC.

**Colonoscopy findings**	***N* (%)**
Colorectal segments	124
**The MVP was assessed using NBI colonoscopy**	
Clear	37 (29.8%)
Obscure	57 (46.0%)
Absent	30 (24.2%)

*MVP, mucosal vascular pattern; NBI, narrow-band imaging*.

### Correlation Between MVP and MES

The relationship between MVP and MES is depicted in Table 3. In the clear pattern group, 89.2% (33/37) of the segments were identified as having an MES of ≤ 1 and 10.8% (4/37) had an MES of 3. In the obscure pattern group, 3.5% (2/57) of the segments were identified as having an MES of ≤ 1, 94.7% (54/57) had an MES of 2, and 1.8% (1/57) had an MES of 3. In the absence of a pattern, 20% (6/30) of the segments were identified as having an MES of 2, and 80% (24/30) had an MES of 3. It was shown that the MVP correlated well with MES (*r* = 0.811, *p* < 0.001) (Table 3).

**Table 3:** Correlation between MVP and Mayo endoscopic score (MES).

**MES/NBI**	**Clear (*n* = 37)**	**Obscure (*n* = 57)**	**Absent (*n* = 30)**	***P*-value**
≤1	33	2	0	0.000
2	0	54	6	
3	4	1	24	

### NBI Findings and Histology

The histological findings from the examined lesions are shown in Table 4. We defined HH as a NI ≤ 1 and histological activity as an NI > 1. Lesions classified as NBI Clear (29/37 showing HH: 78.4%) rarely showed activity on histology. On the contrary, those classified as NBI Obscure (14/57 showing HH: 24.7%) or NBI Absent (0/30 showing HH: 0%) indicated histological activity. There was a significant correlation between NBI findings and histological findings (*r* = 0.614, *p* < 0.001).

**Table 4:** Correlation between the degree of inflammation identified by Nancy index (NI) and endoscopy score.

**Score**	**NI ≤ 1 (*n* = 43)**	**NI > 1 (*n* = 81)**	***P*-value**
**MES**			
0/1	26	9	0.000
2	13	47	
3	4	25	
**NBI**			
Clear	29	8	0.000
Obscure	14	43	
Absent	0	30

*NI, Nancy index; MES; Mayo endoscopic score*.

In the HH group, 60.5% (26/43) of the segments were identified as having an MES of ≤ 1, 30.2% (13/43) had an MES of 2, and 9.3% (4/43) had an MES of 3. In the histological activity group, 11.1% (9/81) of the segments were identified as having an MES of ≤ 1, 58.0% (47/81) had an MES of 2, and 30.9% (25/81) had an MES of 3. There was a significant correlation between WLE classification and histological findings (*r* = 0.471, *p* < 0.001) (Table 4).

### Clinical Relapse in NBI and WLE

During the 12-month follow-up period, 75 patients (60.5%) experienced a clinical relapse. The association between endoscopic activity scores and clinical relapse during the follow-up period is shown in Table 5.

**Table 5:** Clinical relapse by endoscopic score.

**Score**	**Relapse (*n* = 75)**	**No relapse (*n* = 49)**	***P*-value**
**MES**			
0/1	11	24	
2	40	20	0.000
3	24	5	
**NBI**			
Clear	10	27	
Obscure	37	20	0.000
Absent	28	2	

The time to relapse was significantly different between MES in our cohort. In patients with 1-year relapse, 11 patients (14.7%) had an MES of 0/1, 40 patients (53.3%) had an MES of 2, and 24 patients (32.0%) had an MES of 3. However, there was a low correlation between WLE and histological findings (*r* = 0.383, *p* < 0.001). Considering MVP, 10 (13.3%) patients with a clear pattern, 37 patients (49.3%) with an obscure pattern, and 28 patients (37.4%) with an absence of pattern suffered a relapse. There was a significant correlation between NBI and histological findings (*r* = 0.5, *p* < 0.001).

### NBI and WLE and HH in UC

**Figure 3:**
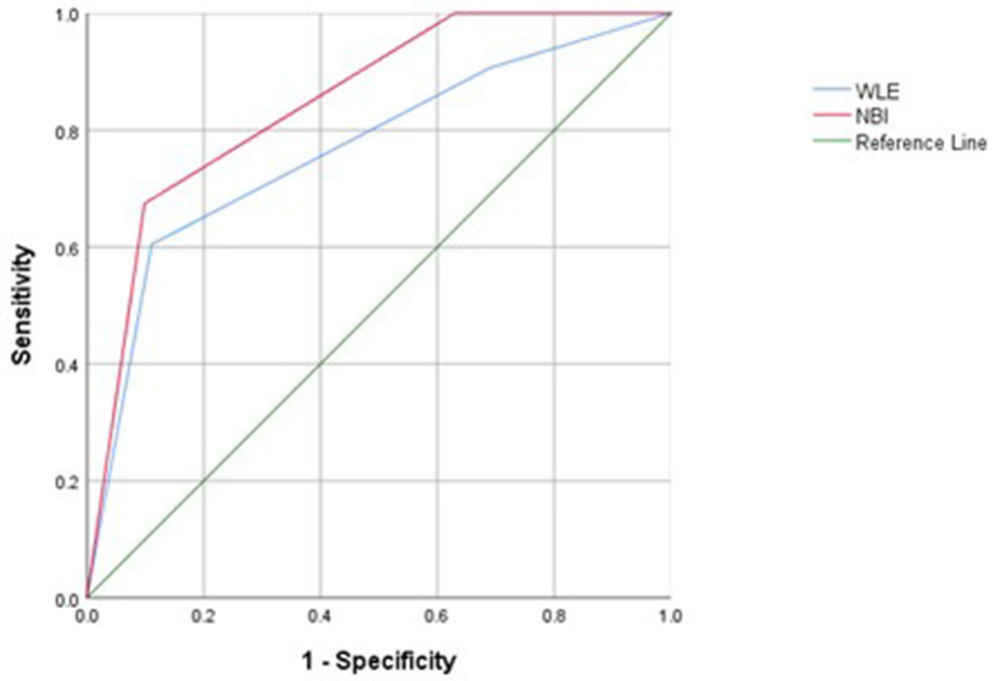
Receiver operating characteristic (ROC) curves of histological healing (HH) assessed with white light endoscopy (WLE) and NBI in patients with UC.

Narrow-band imaging has an AUC of 0.848, a sensitivity of 0.901, a specificity of 0.901, and a 95% confidence interval (*CI*) (0.779–0.971) to predict HH. WLE has an AUC of 0.766, a sensitivity of 0.889, a specificity of 0.605, and a 95% *CI* (0.674–0.859) to predict HH (Table 6, Figure 3). All AUC values were higher than 0.7, suggesting that NBI has a good predictive ability to distinguish the HH group from the other groups.

**Table 6:** Area under the receiver operating characteristic (ROC) curve (AUC), sensitivity, specificity, and Youden index for defined endoscopic and histological healing (HH) in patients with UC.

	**AUC**	**Youden index**	**Sensitivity**	**Specificity**	**95% Confidence interval**
NBI	0.848	0.576	0.901	0.674	0.779–0.917
WLE	0.766	0.494	0.889	0.605	0.674–0.859

### NBI and WLE and Clinical Recurrence in UC

**Figure 4:**
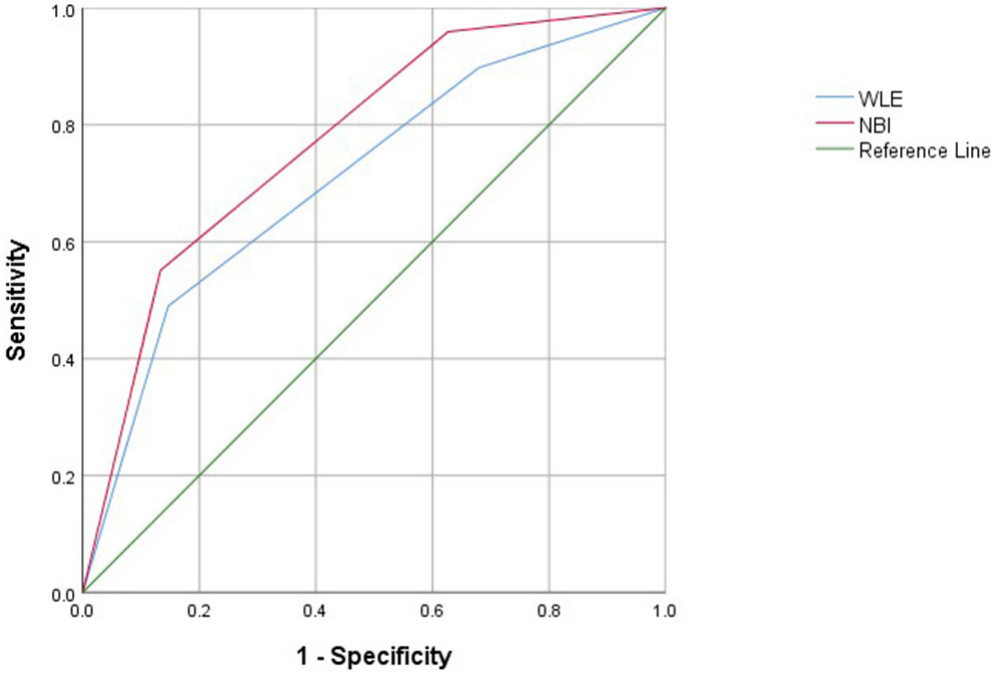
Receiver operating characteristic curves of clinical recurrence assessed with WLE and NBI in patients with UC.

Narrow-band imaging has an AUC of 0.775, a sensitivity of 0.867, a specificity of 0.551, and a 95% *CI* (0.692–0.858) to predict clinical recurrence. WLE has an AUC of 0.710, a sensitivity of 0.853, a specificity of 0.490, and a 95% *CI* (0.616–0.803) to predict HH (Table 7, Figure 4). All AUC values were higher than 0.7, suggesting that NBI has a good predictive ability to distinguish the clinical recurrence group from the other groups.

**Table 7:** Area under an ROC curve, sensitivity, specificity, and Youden index for defined endoscopic and clinical recurrence in patients with UC.

	**AUC**	**Youden index**	**Sensitivity**	**Specificity**	**95% Confidence interval**
NBI	0.775	0.418	0.867	0.551	0.692–0.858
WLE	0.710	0.343	0.853	0.490	0.616–0.803

## Discussion

With the deepening of research and the emergence of various treatment measures, the treatment goal of UC has evolved from clinical remission to endoscopic remission, an absence of inflammatory activity (12). Histological disease activity is considered a predictor of early clinical relapse in patients with UC (13). Therefore, endoscopy plays an important role in determining the severity of inflammation in bowel wall tissue and is critical for monitoring and treating the disease. MES based on WLE assessment is widely used as an objective method to describe the extent of endoscopic activity in UC and has been shown to have a significant overall correlation with histologic severity (14). However, a recent study shows that the endoscopic NBI technique can effectively assess inflammatory activity in UC (15). In comparison with WLE colonoscopy, NBI colonoscopy may be more effective in evaluating locations of inflammation in UC due to greater visualization of the mucosal surface and vascular patterns. At present, it is still uncertain whether MVP based on NBI or MES based on WLE may predict mucosal inflammation and disease recurrence. In our study, we compared the capabilities of NBI colonoscopy with those of WLE for assessing histological activity and prognosis in UC, and the results indicate that NBI provides a better assessment of clinical prognosis and prediction of histology.

Narrow-band imaging is useful in the diagnosis of dysplastic lesions in patients with UC. However, its value for predicting clinical outcomes and HH remains unknown. Sasanuma et al. (16) reported that NBI findings of the colonic mucosa correlated well with histologic activity and discussed their potential to predict endoscopic relapse. In this study, we classified the MVPs of colorectal segments from patients with UC into three pattern types (clear, obscure, or absent) using NBI colonoscopy. The evidence from this study suggests that a clear pattern determined with NBI is a feature of non-recurrence. In patients with a clear pattern, 73.0% (27/37) had no recurrence. Other patterns have a high recurrence rate. In addition, Spearman's correlation analysis suggests a greater degree of correlation between NBI and HH compared with WLE. Because of the strong concordance between NBI and histopathological findings, the number of biopsies can be reduced to some extent, improving the accuracy of biopsies and reducing complications, such as bleeding and perforation.

Our data using NBI of the colonic mucosa suggest that UC relapse may be predicted by vascular changes and mucosal findings. We found that NBI is different from WLE in several respects. Our study showed that NBI is a better predictor of histological activity than WLE. NBI, with an AUC of 0.848 using ROC analysis, has a good capability to predict HH. The sensitivity and specificity of the diagnosing model were 90.1 and 67.4%, respectively, and our study showed that NBI is a better predictor of clinical recurrence than WLE. NBI, with an AUC of 0.775 using ROC analysis, has a good capability to predict clinical recurrence. Therefore, we conclude that NBI correlates with HH and could be a better predictor of clinical recurrence than WLE. The vascular changes and mucosal findings may be due to active inflammation; NBI can better assess vascular change and mucosal findings.

This study has some limitations. First, the sample size is small and from a single center. Second, the follow-up period in patients with UC was relatively short. Further, the correlation between NBI and other endoscopy scores, such as Ulcerative Colitis Endoscopic Index of Severity (UCEIS), was not analyzed. Studies need to be carried out to validate NBI-predicted clinical recurrence in patients with an MES of 1 or 0. To further corroborate our results, prospective multicenter studies involving larger samples, lesions with dysplasia, and comparison of patients with UC are required.

In conclusion, the MVP based on NBI colonoscopy correlates well with the MES based on WLE in patients with UC. Moreover, MVP determined with NBI is a more useful method for predicting HH and clinical recurrence than MES determined with WLE in patients with UC. In the future, NBI will be performed even in patients with normal mucosa on endoscopy, especially in cases of vascular obscuration.

## Data Availability Statement

The raw data supporting the conclusions of this article will be made available by the authors, without undue reservation.

## Ethics Statement

The studies involving human participants were reviewed and approved by Weifang People Hospital. The patients/participants provided their written informed consent to participate in this study. Written informed consent was obtained from the individual(s) for the publication of any potentially identifiable images or data included in this article.

## Author Contributions

TH: data curation and writing-original draft preparation. LZ: conceptualization and methodology. PP: data sorting. SS: paper revision. HQ: funding acquisition. All authors contributed to the article and approved the submitted version.

## Conflict of Interest

The authors declare that the research was conducted in the absence of any commercial or financial relationships that could be construed as a potential conflict of interest.

## Publisher's Note

All claims expressed in this article are solely those of the authors and do not necessarily represent those of their affiliated organizations, or those of the publisher, the editors and the reviewers. Any product that may be evaluated in this article, or claim that may be made by its manufacturer, is not guaranteed or endorsed by the publisher.
